# Enteral Nutrition for Feeding Severely Underfed Patients with Anorexia Nervosa

**DOI:** 10.3390/nu4091293

**Published:** 2012-09-14

**Authors:** Maria Gabriella Gentile

**Affiliations:** Clinical Nutrition, Eating Disorders Unit, Niguarda Hospital, Milan 20122, Italy; Email: mariagabriella.gentile@ospedaleniguarda.it; Tel.: +39-02-6444-2839; Fax: +39-02-6444-2593

**Keywords:** feeding regimens, extreme undernutriton, refeeding syndrome, enteral feeding, anorexia nervosa, hypophosphoremia

## Abstract

Severe undernutrition nearly always leads to marked changes in body spaces (e.g., alterations of intra-extracellular water) and in body masses and composition (e.g., overall and compartmental stores of phosphate, potassium, and magnesium). In patients with severe undernutrition it is almost always necessary to use oral nutrition support and/or artificial nutrition, besides ordinary food; enteral nutrition should be a preferred route of feeding if there is a functional accessible gastrointestinal tract. Refeeding of severely malnourished patients represents two very complex and conflicting tasks: (1) to avoid “refeeding syndrome” caused by a too fast correction of malnutrition; (2) to avoid “underfeeding” caused by a too cautious rate of refeeding. The aim of this paper is to discuss the modality of refeeding severely underfed patients and to present our experience with the use of enteral tube feeding for gradual correction of very severe undernutrition whilst avoiding refeeding syndrome, in 10 patients aged 22 ± 11.4 years and with mean initial body mass index (BMI) of 11.2 ± 0.7 kg/m^2^. The mean BMI increased from 11.2 ± 0.7 kg/m^2^ to 17.3 ± 1.6 kg/m^2^ and the mean body weight from 27.9 ± 3.3 to 43.0 ± 5.7 kg after 90 days of intensive in-patient treatment (*p* < 0.0001). Caloric intake levels were established after measuring resting energy expenditure by indirect calorimetry, and nutritional support was performed with enteral feeding. Vitamins, phosphate, and potassium supplements were administered during refeeding. All patients achieved a significant modification of BMI; none developed refeeding syndrome. In conclusion, our findings show that, even in cases of extreme undernutrition, enteral feeding may be a well-tolerated way of feeding.

## 1. Introduction

Severe undernutrition nearly always leads to marked changes in body spaces (e.g., alterations of intra-extracellular water) and in body masses and composition (e.g., overall and compartmental stores phosphate potassium, magnesium). Hemodynamic instability, severe volume derangement, electrolytic disturbances, hypoglycaemia, hypothermia and bone marrow depression are common in severe undernutrition [1]. If the caloric deficiency is severe and enough prolonged adults can lose up to half of their body weight, and body mass index decreases to 13 kg/m^2^ or less. Severe undernutrition affects every body area: digestive system, cardiovascular and respiratory systems, reproductive system, nervous system, muscle, blood, metabolism and immune system [2,3,4,5,6,7]. 

Nutritional support can be regarded as a graded process developing through different levels which are not necessarily to be considered mutually exclusive: (1) improving energy and nutrient intake from ordinary food; (2) oral nutritional support (sip feed); (3) artificial nutrition choosing enteral nutrition (EN) as preferred route of feeding if there is a functional, accessible gastrointestinal tract [8,9,10,11,12].

Refeeding of severely malnourished patients represents two very complex and conflicting tasks:

1. avoid “refeeding syndrome” caused by a too fast correction of malnutrition [13,14,15];
2. avoid “underfeeding” caused by a too cautions rate of refeeding.

These patients suffer from poor myocardial contractility and circulatory volume should be evaluated with care. Electrolyte disturbances and vitamin deficiencies are quite frequent, and during refeeding these derangements can actually arisen or aggravate. 

Nutritional support in severely undernourished patients has main objectives: (1) to restore lean body mass; (2) to preserve or restore immune function; (3) to contrast or avert metabolic complications; (4) to attenuate oxidative cellular injury and metabolic response to stress or starvation; (5) to prevent heart failure and respiratory failure. 

Caring for severely starved patients and re-starting nutrition requires close monitoring seeking for early signs or symptoms of refeeding syndrome and a specialized care plan.

In the more complicated cases, *i.e.*, patients with extreme undernutrition and life threatening weight loss or patients unable or unwilling to consume an adequate oral diet, there is the indication to start artificial nutrition. 

To avoid “refeeding syndrome” and to avoid “underfeeding” of these critical and severely undernourished patients, the caloric intake should be planned starting with indirect calorimetric measurements, because resting energy expenditure (REE) is the main component of daily expenditure particularly in severely undernourished patients [16,17,18].

If this measurement is not possible, we should estimate energy needs with the Harris Benedicts formula considering that in patients without inflammatory complications the energy need is 70–80 percent in respect to the estimated value [18]. 

Although there is a general consent regarding crucial nutritional rehabilitation, there are only few studies that report artificial nutrition in severe undernutrition. Given the recognized difficulties in conducting randomized clinical trials in these critical patients, evidence-based guidelines for use of enteral or parenteral nutrition are lacking.

In order to make a choice, we should consider the two following points:

1. The international guidelines on the use of artificial nutrition state that “if the gut works you must use it”.
2. In the majority of critically ill patients the preferred way of feeding is EN, because it is safer compared to parenteral nutrition as it is well documented in numerous prospective randomized controlled trials involving the effects of critical illness on mortality. The most consistent outcome effect of EN is a reduction in infectious morbidity, a positive impact on the duration of treatment and a normalization of the endocrine and metabolic status [19].

The aim of this paper is to discuss the modality of refeeding severe undernourished patients by EN and to present our experience with the use of enteral tube feeding for the gradual correction of very severe undernutrition while avoiding the refeeding syndrome.

## 2. Patients and Methods

### 2.1. Patients

Out of 89 inpatients referred to the Niguarda Hospital Eating Disorders Unit between December 2009 and February 2012, affected by undernutrition due to anorexia nervosa (AN) (according to the Diagnostic and Statistical Manual of Mental Disorders) [20] we identified 10 patients who were considered eligible for the study, as they presented a BMI ≤ 12 kg/m^2^ upon our hospital admission and underwent at least 90 days of observation.

### 2.2. Measurements

Anthropometry and resting metabolic rate. Body weight was recorded to the nearest 100 g using a standard physician's weight scale with the patient wearing only underwear (without shoes). Height was determined to the nearest 0.5 cm on a standard stadiometer. 

Resting metabolic rate was estimated by indirect calorimetry. Resting gas exchange was measured by open circuit, indirect calorimetry (Sensor Medics) for 30 min. Before each measurement, the system was re-calibrated using a reference gas mixture of 95% O_2_ and 5% CO_2_.

Blood samples were usually taken by an antecubital vein puncture. Hematological parameters and a biochemical assessment were determined by the hospital laboratory. And they were performed using published methods. Some laboratory tests were repeated daily. We report data on days 0, 15, 30, 60, and 90. Plasma electrolytes (especially phosphate, sodium, potassium and magnesium), glucose levels and any deficiencies were closely monitored and corrected during feeding. 

### 2.3. Nutritional Rehabilitation and Medical Treatment

Caloric intake levels were established beginning with indirect calorimetric measurements.

In the patients in a life-threatening state, immediate nutritional support with EN was begun at a low rate with temporary nasogastric feeding, closely monitored and regulated via an electronically operated pump. Nasogastric feeding was the preferred procedure because it is a safe and simple non-surgical procedure.

The patients that were not affected by any specific disease (such as renal or hepatic insufficiency or diabetes), it was possible to use a polymeric diet free of lactose and gluten, in the form of a high nitrogen, complete fluid formula.

In order to reduce gastric discomfort and to avoid fluid overload, we used a high content of calories (1.7–2 kcal/mL) unless the patients presented with plasmatic hypoonchia. No interruption of oral feeding, if it was accepted, was done.

Dieticians helped patients to choose their own meals and provided a personalized meal plan.

To prevent hypoglycemic episodes, we used a combination of continuous EN and intravenous fluid 10% glucose (20–40 mL/h) during 24 h [18]. 

Vitamin-thiamine and B vitamins were always supplemented before to start re-alimentation and they were continued during the refeeding period. We started oral phosphate supplements or/and i.v. NaPhos even before a complete serum electrolyte panel was available.

The phosphate dose was evaluated in each patient. The amounts were highly variable and required strict, daily monitoring of serum phosphate. The range of phosphate supplementation was from 80 to 1000 mg/day [21,22,23]. Potassium and magnesium were added according to blood levels. Body weight was checked at least once a day. The need of water is usually of 25–30 mL/kg/day.

## 3. Data Analysis

Data were analyzed with the SAS PACKAGE (Release 8.2 by SAS Institute Inc., Cary, NC, USA, 2002). The results are reported as medians and/or means ± standard deviations and ranges. 

Changes in body weight and in body mass indexes between admission and after EN treatment were tested with the sign test for paired data. The hypothesis of no difference between the body weight and the body mass index at admission and after EN treatment was rejected by a *p* < 0.0001 for both variables.

## 4. Results

Ten patients were eligible for the study with a mean BMI of 11.2 ± 0.7 kg/m^2^ (range 9.6–11.9 kg/m^2^) and a mean age of 23.9 ± 11.1 years.

An overview of the patients, demographic and clinical characteristics is given in Table 1. As a consequence of extreme undernutrition, all patients presented with low blood pressure, apathy and irritability, reduced muscle size, hypothermia and a clinically significant reduction in measured REE (−37.0 ± 8.8 percent) and estimated basal metabolic rate according to the Harris Benedict formula.

They did not present other medical complications except those that were a consequense of severe undernutrition. A tube was inserted by a nurse as soon as possible—usually within 1–2 h after admission into the Unit. During the first weeks, nasogastric tube feeding was continuously delivered over 24 h daily, using feed pumps. 

All patients had an anticubital vein access placed for an i.v. infusion of 10% glucose fluids in prophylactic way to prevent hypoglycemia. All patients were supplemented from the first hours with thiamine, a full dose of other B vitamins, and oral phosphate (KPhos) supplements. In three patients we added intravenous administration of NaPhos to maintain the phosphate within the normal range in the plasma. The treatments are summarized in Table 2.

**Table 1:** Antropometric demographic and clinical data in 10 anorexia nervosa patients with extreme undernutrition ^a^.

Time	Day 0	15 days	30 days	60 days	90 days
No. of patients	10				
Age	23.9 ± 11.1 (11.7–48.7)				
Body weight (kg)	27.9 ± 3.3 (22.5–32.2)	32.0 ± 3.8 (26.3–36.5)	34.9 ± 3.5 (30.0–41.1)	39.1 ± 4.8 (30.5–44.6)	43.0 ± 5.7 (31.0–50.0)
Height (cm)	157 ± 0.1 (145–167)				
BMI ^b^ (kg/m^2^)	11.2 ± 0.7 (9.6–11.9)	12.9 ± 0.9 (11.4–14.5)	14.1 ± 0.9(13.2–15.6)	15.8 ± 1.3 (14.1–17.9)	17.3 ± 1.6 (13.9–20.0)
Estimated resting metabolic rate (kcal/24 h)	1092 ± 115 (809–1197)				
Measured resting metabolic rate (kcal/24 h)	739 ± 161 (514–1123)				
% difference *versus* estimated basal metabolic rate according to Harris Benedict formula	−37.0 ± 8.8 (−53 to −26)				
Age of onset/diagnosis of anorexia nervosa (years)	19.3 ± 8.9 (10.0–39.2)				
Duration of disease (months)	56.3 ± 47.7 (3.0–131.0)				
Age at beginning of treatment at our Unit (years)	24.0 ± 10.6 (11.7–48.7)				
Amenorrhoea (No.)	9				
Prepuberal (No.)	1				

^a^ Values are means ± SDs (range); ^b^ BMI = Body Mass Index.

**Table 2:** Treatment of 10 anorexia nervosa patients with extreme undernutrition ^a^.

Time	Day 0	15 days	30 days	60 days	90 days
Enteral feeding regimen (kcal/day)	450.0	837.5	1100.0	1000.0	800.0
Amount of enteral fluid diet (mL/day)	300.0	650.0	600.0	500.0	400.0
Caloric density (kcal/mL)	2.0	2.0	2.0	2.0	2.0
Protein (enteral feeding) (g/kg body weight)	0.7	1.3	1.5	1.2	0.9
Protein (oral diet) (g/kg body weight)	1.3	1.5	1.7	1.8	1.8
Protein (total)(g/kg body weight)	2	2.8	3.0	2.9	2.6
Fiber (enteral feeding) (g)	5.0	11.0	12.5	12.5	10.0
Δ Body weight ^b^ (kg)		4.35	7.3	11.95	16.7
Phosphatei.v. ^c^/oral (mg/day)	500.0	500.0	375.0	250.0	250 (1) ^e^
Glucose ^d^ i.v. infusion (kcal/day)	200.0	400.0 (9) ^e^	400.0 (4) ^e^	200.0 (1) ^e^	200 (1) ^e^
Oral diet (kcal/day)	408	1204	1412	1819	2135
Enteral feeding regimen + Glucose (kcal/day)	800.0	1200.0	1200.0	1000.0	800.0
Oral diet + enteral feeding regimen + Glucose (kcal/day)	1199	2508	2784	2935	2871

^a^ Values are medians; ^b^ Δ Body weight = increase of body weight starting from time = 0; ^c^ i.v. = intravenous; ^d^ Glucose infused by intravenous fluid (10%) during 24 h; ^e^ ( ) number of subjects; if the number is not indicated, it is understood as being 10 patients; As primary outcome we obtained a significant modification in body weight from 27.9 ± 3.3 to 43.0 ± 5.7 kg and of BMI from 11.2 ± 0.72 kg/m^2^ to 17.3 ± 1.6 kg/m^2^ (*p* < 0.0001).

The amount of calories from EN plus glucose infusion was 30.2 ± 9.6 kcal/BW/day on 0 day; 41.5 ± 18 kcal/BW/day on day 15 and 31.5 ± 15 kcal/BW/day on day 30, then it progressively decreased. 

The estimated amount of calories from oral diet (common food) gradually increased from 14.3 ± 12 kcal/BW/day on 0 day to 43.9 ± 12 kcal/BW/day on day 30 and 48.5 ± 10 kcal/BW/day on day 90. These amounts may have been overestimated based on the data referred by patients. In any case, we must also consider that 105,910 extra calories were needed to reach an extra weight gain of 15.1 kg in 90 days, which these patients achieved.

As secondary outcomes we carefully prevented critical electrolyte derangements, *i.e.*, hypophosphatemia and hypokalemia. We did not observe any major signs and symptoms of refeeding (heart failure, edema, rhabdomyolysis and encephalopathy). Table 3 shows the mean values and standard deviation of the hematological parameters and biochemical values of the 10 patients during the 90-day treatment period. 

**Table 3:** Hematological parameters and biochemical values of 10 anorexia nervosa patients, monitored frequently during refeeding treatments ^a^.

Time	Day 0	15 days	30 days	60 days	90 days
Red blood cells (10^9/L)	3.8 ± 0.7 (2.1–4.4)	3.1 ± 0.6 (2.4–4.2)	3.4 ± 0.5 (2.7–4.2)	3.9 ± 0.5 (3.2–4.8)	4.1 ± 0.4 (3.9–5.0)
Hemoglobin (g/dL)	12.2 ± 2.2 (7.6–14.8)	10.0 ± 1.4 (8.2–12.6)	11 ± 1.5 (8.8–13.5)	12.2 ± 1.0 (10.6–13.6)	12.7 ± 0.8 (11.6–14.0)
Hematocrit (%)	36.2 ± 6.5 (22.1–43.6)	30.6 ± 4.6 (25.4–38.5)	33 ± 4.2 (27.1–40.1)	37.2 ± 3.6 (31.6–41.8)	38.4 ± 2.6 (34.5–42.9)
White blood cells (10^9/L)	5.0 ± 4.0 (2.0–15.9)	5.0 ± 1.6 (2.1–7.9)	6 ± 2.3 (2.6–9.3)	6.1 ± 2.0 (3.8–10.3)	5.8 ± 1.7 (3.5–9)
Platelets (10^9/L)	214.2 ± 99.1 (60–381)	340.3 ± 200.1 (188–836)	283 ± 123.9 (153–550)	250.3 ± 68.4 (158–382)	247.3 ± 42.7 (177–309)
Lymphocytes (10^9/L)	1.8 ± 1.2 (0.5–4.8)	1.4 ± 0.6 (0.9–2.5)	1.6 ± 0.6 (0.8–3.0)	1.7 ± 0.6 (0.9–3.0)	1.7 ± 0.4 (1.1–2.4)
Glucose (mg/dL)	82.7 ± 17.5 (56–110)	74.9 ± 6.2 (63–84)	75 ± 13.1 (60–95)	64.7 ± 15.0 (61–100)	73.0 ± 19.1 (38–108)
Sodium (mmol/L)	140.4 ± 2.8 (136–145)	142.4 ± 0.8 (141–144)	143 ± 1.7 (141–147)	142.6 ± 1.4 (141–145)	141.8 ± 2.3 (139–146)
Potassium (mmol/L)	4.2 ± 0.4 (3.5–4.6)	4.4 ± 0.3 (4.0–4.8)	4.3 ± 0.6 (3.7–5.6)	4.4 ± 0.4 (3.9–4.9)	4.4 ± 0.4 (3.9–5.1)
Chloride (mmol/L)	98.5 ± 5.3 (91–106)	103.7 ± 2.1 (101–107)	104.2 ± 2.7 (98–108)	101.9 ± 2.7 (98–105)	102.9 ± 2.7 (98–107)
Bicarbonate (mmol/L)	29.0 ± 3.5 (25–35)	27.2 ± 2.4 (23–31)	26.6 ± 2.8 (23–31)	25.1 ± 2.8 (20–30)	26.3 ± 2.8 (22–30)
Calcium (mg/dL)	9.1 ± 0.4 (8.4–9.6)	8.8 ± 0.5 (8.2–9.5)	9.2 ± 0.3 (8.6–9.7)	9.5 ± 0.7 (8.7–10.7)	9.5 ± 0.5 (8.8–10.6)
Phosphorus (mg/dL)	3.4 ± 0.7 (2.2–4.4)	4.2 ± 0.5 (3.6–4.9)	4.5 ± 0.4 (3.8–5.0)	4.6 ± 0.4 (4.1–5.2)	4.4 ± 0.5 (3.8–5.4)
Magnesium (mEq/L)	1.7 ± 0.1 (1.4–1.9)	1.6 ± 0.1 (1.3–1.7)	1.6 ± 0.1 (1.4–1.8)	1.6 ± 0.2 (1.3–1.8)	1.6 ± 0.2 (1.2–1.9)
Aspartate aminotransferase (U/L)	48.6 ± 31.9 (17–107)	31.3 ± 14.0 (18–56)	30.9 ± 13.2 (12–54)	30.7 ± 9.6 (16–44)	26.5 ± 6.5 (18–41)
Alanine Aminotrasferase (U/L)	79.4 ± 63.2 (16–188)	55.4 ± 25.2 (18–103)	51.5 ± 26.6 (18–85)	42.7 ± 25.0 (15–92)	30.3 ± 13.0 (19–63)
γ-Glutamyl transferase (U/L)	52.0 ± 37.8 (5–120)	46.7 ± 28.1 (7–96)	40.4 ± 26.6 (7–84)	31.2 ± 27.3 (7–90)	23.4 ± 17.5 (7–51)

^a^ Values are means ± SDs and ranges in brackets.

During the whole observation period, we observed only one episode of severe hypoglycemia (glucose of 38 mg/dL) on the 90th day of treatment when the patient was treated only with an oral diet.

Hypophosphatemia defined by a phosphate value <3 mg/dL was observed in three patients before starting treatment (day = 0), and never during the 90 days of therapy. Blood potassium and magnesium values were always maintained in normal ranges.

## 5. Discussion

In severely undernourished patients, the goal of nutritional rehabilitation should be to obtain a weekly weight gain of 0.5–1 kg, which means an excess of around of 3500–7000 kcal a week [24]. The average weekly weight gain in our extremely undernourished patients was 1.1 kg/week as suggested by the literature [24,25]. The continuous monitoring of fluid intake was necessary in order to avoid fluid overload with consequent refeeding edema and cardiac failure. This was achieved by weighting the patients at least once a day, checking tissues for edema daily, and monitoring water intake by mouth.

Metabolic complications were prevented and/or minimized by careful monitoring. Special care is necessary to prevent hypoglycemia, which is virtually always possible because the liver lacks sufficient substrate to maintain the patient’s glycemia. In our experience, the combined use of continuous EN and continuous intravenous fluid with 10% glucose during 24 h prevented hypoglycemic episodes. Other authors have reported that hypoglycemia occurred in almost half of their patients with severe anorexia [26]. Liver function was well maintained as shown by liver function test otherwise of other authors [26,27]. Furthermore, an approach of continuous feeding may avoid wide glucose and insulin excursions and minimize refeeding shifts as well as anxiety and nervousness [28].

We choose to start with a 24 h continuous supplementation of EN to reduce gastric discomfort, diarrhea, and metabolic alterations, only when the undernutrition was partially corrected, die we gradually reduce the infusion time. Having the relevant skills and trained nurses prevents complications with the initial placement of the tube for feeding, its obstruction and microbial contamination by enteral feeding.

EN was well tolerated and the patients with life-threatening undernutrition did not suffer any major discomfort, such as nausea, vomiting or diarrhea. The Espen guidelines on enteral nutrition indicate that patients with severe undernutrition should receive EN up to a total of 25–30 kcal/kg BW/day. If these target values are not reached, supplementary parenteral nutrition should be given [9], which was not necessary with our patients. Adequate hydration and the use of a fiber-containing formula prevented or treated constipation, in some of these cases we also used probiotic supplementation. No mechanical complications such as aspiration or tube malposition were observed.

We choose to use EN because we considered parenteral nutrition to be a higher risk for the development of refeeding syndrome, and we agreed with international guidelines, which consider the use of parenteral nutrition instead of EN not necessary for nutritional rehabilitation in patients without gastrointestinal dysfunction [9]. 

An empathic approach by the whole multidisciplinary team helped to obtain a reasonable compliance and co-operation; behavioral and psychological therapy for patient and their parents were always put into place [18].

## 6. Conclusions

Given the recognized difficulties in conducting randomized clinical trials in patients with AN, particularly if they are in life-threatening situations, evidence-based guidelines for enteral or parenteral nutrition are lacking.

Slow initiation of caloric intake requires daily management to prevent refeeding complications such as hypoglycemia and hypophosphoremia during the body’s conversion from a catabolic to an anabolic state, gastroparesis and slowed colonic transit, characteristics of AN patients, are not a contraindication to use enteral feeding if adequate protocols are used.

Nutritional rehabilitation of severely anorexic patients is a highly important component of a patient’s care, but it represents a very complicate task, because rapid refeeding can result in a serious sequence of events, as was shown in a French multicenter study about AN patients treated in intensive care units, which reported a crude mortality of 10 percent [27], and a very high percentage of metabolic, hepatic, and cardio-respiratory complications [26,27,28].

Our findings show that, even in cases of extreme undernutrition, enteral feeding may be considered a well-tolerated treatment of artificial nutrition. In addition, we support that the prophylactic supplementation of phosphorous and potassium during the first weeks of refeeding is effective in preventing hypophosphatemia and other electrolyte derangements [21,23] while others recommended close monitoring and supplementation when indicated [28] but they experienced a higher presence of hypophosphoremia that is often recognized as a hallmark for refeeding syndrome.

Weight restoration of AN patients is an essential part of treatment, without it, AN patients may face serious and even fatal medical complications. Undernutrition-induced cognitive deficits may interfere with psychological intervention. Although nutritional management is a fundamental components of treatment, current practice is based more on experience and consensus than on published evidence, particularly regarding really sick AN patients, where prevention and early-detection of refeeding syndrome is a key point, but avoid underfeeding it is equally mandatory.
